# A chionodracine-derived peptide, KHS-Cnd, as an anti-virulence agent against multidrug-resistant *Acinetobacter baumannii* clinical strains

**DOI:** 10.3389/fcimb.2025.1526246

**Published:** 2025-02-14

**Authors:** Marco Artini, Irene Paris, Esther Imperlini, Francesco Buonocore, Gianluca Vrenna, Rosanna Papa, Laura Selan

**Affiliations:** ^1^ Department of Public Health and Infectious Diseases, Sapienza University, Rome, Italy; ^2^ Department for Innovation in Biological, Agro-Food and Forest Systems, University of Tuscia, Viterbo, Italy; ^3^ Research Unit of Diagnostical and Management Innovations, Children’s Hospital and Institute Research Bambino Gesù, Rome, Italy

**Keywords:** *Acinetobacter baumannii*, biofilm, twitching motility, surface-associated motility, antimicrobial peptide

## Abstract

About 71% of healthcare-associated infections are due to antibiotic-resistant bacteria, such as carbapenem-resistant *A. baumannii*, classified by World Health Organization into a critical priority group of pathogens. The antimicrobial resistance profile of *A. baumannii* relies on its ability to produce several virulence factors, including biofilm formation. Its ability to adhere and persist on surfaces as biofilm has contributed to its pathogenicity and drug resistance. In this study, the ability of an antimicrobial peptide (a chionodracine-derived peptide named KHS-Cnd) to inhibit or reduce biofilm formation was investigated as an example of a potential strategy to counteract infections caused by biofilm-forming pathogens. To this aim, the antimicrobial profiles were first analyzed in selected *A. baumannii* strains, two reference and six clinical strains, all biofilm-forming with different capability, regardless of whether they are drug resistant or sensitive. Successively, we investigated the bactericidal activity of the peptide that showed MIC values ranging from 5 to 10 µM and a significative antibiofilm activity on all tested strains at sub-inhibitory concentrations. In fact, KHS-Cnd can hinder biofilm *A. baumannii* strains formation with an inhibition percentage ranging between 65% and 10%. Also a statistically significant reduction of mature biofilm ranging from 20% to 50% was observed in four out of eight tested *A. baumannii* strains. KHS-Cnd impacts various stages of biofilm formation, including the inhibition of surface-associated and twitching motilities depending on the different strain. In particular, our results showed that only two strains possessed surface-associated motility that was strongly impaired by KHS-Cnd treatment; three clinical strains, instead, showed twitching motility, whose inhibition for two of them was evident after 24 h of incubation with peptide. Moreover, the invasion of pulmonary cells by *A. baumannii* was significantly impaired with a reduction of about 32% after treatment with 1.25 µM KHS-Cnd. Finally, when the peptide was used together with ceftazidime/avibactam against resistant *A. baumannii* strains, it was able to reduce the minimal inhibitory concentration of antibiotics needed to inhibit the microorganism growth.

## 1 Introduction

On May 17, 2024, the World Health Organization (WHO) published an updated list of antibiotic-resistant bacteria responsible for nosocomial infections, mainly related to respiratory, urinary and gastro-intestinal tracts (Sati et al., 2024). Based on the recent surveillance data, WHO grouped 24 bacterial species across 15 families of antibiotic-resistant pathogens into critical, high and medium categories, thus providing information on therapeutic strategies to counteract the antimicrobial resistance (AMR) (Jesudason, 2024).

According to the European Centre for Disease Prevention and Control (ECDPC) more than 3.5 million cases of healthcare-associated infections (HAIs) are estimated leading to more than 90,000 deaths each year. These HAIs represent 71% of infections cases with antibiotic-resistant bacteria, including bacteria resistant to antibiotics of last resort, such as carbapenem-resistant *Acinetobacter baumannii*, classified by WHO into the critical priority group (Sati et al., 2024; European Centre for Disease Prevention and Control, 2024).

*A. baumannii*, an aerobic Gram-negative bacterium belonging to ESKAPE pathogens, has been isolated from infected patients (Nocera et al., 2021; Pustijanac et al., 2023) and different natural sources (such as water and soils) (Denissen et al., 2022) thus demonstrating a great ability to adapt to hostile environments and survive on biotic and abiotic surfaces. *A. baumannii* clinical isolates were reported to be genetically similar to environmental ones, but with different antibiotic susceptibility (Ying et al., 2015). These strains exhibit a broad range of intrinsic mechanisms of resistance to different antibiotics such as beta-lactams, carbapenems, fluoroquinolones and aminoglycosides, thus hampering the treatment effectiveness of a wide spectrum of infections, including pneumonia (Dahdouh et al., 2016; Castilho et al., 2017; Hansen et al., 2023).

The heterogenic AMR profile of *A. baumannii* relies on its ability to produce several virulence factors, including biofilm formation (Ganesh et al., 2024). The propensity of *A. baumannii* to adhere and persist on surfaces in biofilm phenotype has contributed to its pathogenicity and drug resistance (Eze et al., 2018; Artini et al., 2023; Marino et al., 2024).

Biofilm is a three-dimensional structure of microbial communities, whose formation is a dynamic process that allows *A. baumannii* persistence on a biological surface (such as host mucosal tissue) or inanimate surface (like medical devices and implants). In a biofilm, in fact, bacteria are surrounded by a self-produced matrix enriched with exopolysaccharides, proteins and extracellular DNA (eDNA) (Pakharukova et al., 2018). Since this robust matrix establishes a protective layer, bacterial biofilm cells evade the host immune responses and resist conventional antibiotics (Choudhary et al., 2022; Mendes et al., 2023).

As the rise of multidrug-resistant *A. baumannii* strains has become a public global concern, new antimicrobial strategies are urgently needed. Hence, the development of antimicrobial drugs able to control/inhibit biofilm formation with or without impacting bacterial viability could represent a promising solution to AMR. In this context, antimicrobial peptides (AMPs) have been considered as a valid alternative to conventional antibiotics due to their low propensity to induce AMR and to be not toxic for the host (Luo et al., 2023; Imperlini et al., 2023). These natural peptides, usually long up to 50 amino acids in their bioactive form and mostly cationic, exhibit a broad spectrum of antimicrobial activities against bacteria, viruses, yeasts and protozoa (Hassan et al., 2022; Ajose et al., 2024).

In this study, after characterizing AMR profile and phenotypic features of six clinical and two reference *A. baumannii* strains, their ability to form biofilm and their motility were analyzed; then, the effects of AMP on these processes were evaluated. The tested peptide was a mutant derived from the natural chionodracine (Cnd) peptide identified in the Antarctic fish *Chionodraco hamatus*, named KHS-Cnd, that previously showed highest capacity to kill ESKAPE pathogens (Olivieri et al., 2018; Buonocore et al., 2019) and the ability to impair biofilm formation in *Pseudomonas aeruginosa* clinical isolates and to reduce their invasion in human pulmonary cells (Artini et al., 2022).

The main rationale of this study is to investigate the peptide’s ability to inhibit or reduce biofilm formation, which is associated with the virulence and persistence of microbial infections. By targeting biofilm production, the peptide could offer a potential strategy for combating infections caused by biofilm-forming pathogens.

## 2 Materials and methods

### 2.1 Bacterial strains and growth conditions

Six clinical and two reference strains of *A. baumannii* were used. Reference ATCC 17978 strain was firstly isolated in 1951 from a fatal case of meningitis in a 4-month-old patient in France (Eijkelkamp et al., 2014). Reference ATCC 19606 strain was isolated in the mid-1980s from a urine sample in the United States of America (Eijkelkamp et al., 2014). Clinical strains of *A. baumannii* were isolated from respiratory infections from patients admitted to Pediatric Hospital and Institute of Research Bambino Gesù (OPBG) in Rome, Italy (OPBG).

Bacteria were grown in Brain Heart Infusion broth (BHI, Oxoid, Basingstoke, UK). Bacterial cells were grown in planktonic condition at 37°C under orbital shaking (180 rpm), while biofilm formation was performed at 37°C in static conditions.

### 2.2 Peptide

The peptide KHS-Cnd (WFGKLYRGITKVVKKVKGLLKG) was synthesized by Caslo Aps (Caslo Aps Kongens, Lyngby, Denmark) with a grade of 98% purity. KHS-Cnd was solubilized in PBS at a concentration of 400 µM and stored at -20°C until use.

### 2.3 Determination of minimal inhibitory concentration and minimal bactericidal concentration

*A. baumannii* strains were classified based on their antimicrobial resistances, according to The European Committee on Antimicrobial Susceptibility Testing (http://www.eucast.org, 20th October 2023). To evaluate the sensitivity or resistance of *A. baumannii* strains to different antibiotics, two techniques were used. The Kirby-Bauer test was performed in all cases except for colistin for which the diffusion and dilution method was selected. The strains are defined as wild-type (WT) if they are sensitive to all antimicrobials, while multidrug-resistant (MDR) if they are resistant to at least one agent in three or more antimicrobial categories and pandrug-resistant (PDR) if they are resistant to all antibiotics in all classes.

MIC and MBC values for KHS-Cnd were determined by liquid growth inhibition assays using serial dilutions of the peptides in a sterile 96-well polystyrene flat-based plate. The peptide was dissolved in sterile PBS and diluted in Mueller Hinton broth (MHB) (Oxoid, Basingstoke, UK) to reach a final concentration of 20 μM. Overnight bacterial cultures were diluted 1:100 in BHI medium. Each well contained 100 μL of bacterial suspension and KHS-Cnd serially diluted starting from a concentration of 20 μM. The MIC value is the lowest concentration of the peptide that completely inhibited growth after 24 h of incubation at 37°C in static conditions. To determine the MBC vale, we plated 10 μL from the wells with no visible microbial growth onto Tryptone soy agar (Oxoid, Basingstoke, UK) plates and incubated them for 24 h at 37°C. All tests were performed in triplicate in two different experimental sessions, and for each series of experiments, positive control (without peptide) and negative control (without bacteria) were included.

### 2.4 Biofilm formation

The biofilm content was quantified by the microtiter plate (MTP) biofilm assay (Artini et al., 2023). From an overnight grown bacterial culture, a 1:100 dilution was added in the wells of a sterile 96-well polystyrene flat-based plate prefilled with medium in the presence and in the absence of KHS-Cnd at a concentration corresponding to 1/4 of MIC value. The plates were incubated overnight at 37°C under static conditions. After incubation, the supernatant containing planktonic cells was gently removed and the plate was washed with distilled water. Then, the plate was dried in the inverted position. Staining was performed with 0.5% (w/v) crystal violet for 15 min at room temperature. The excess crystal violet was carefully removed; the plate was washed again with distilled water and dried to quantify the biofilm formation. The biofilm was solubilized with 20% (v/v) glacial acetic acid and 80% (v/v) ethanol, and spectrophotometrically quantified at 590 nm. Each data point was composed of three independent experiments, each performed in at least three replicates.

### 2.5 Mature biofilm

Assays on preformed biofilm were also performed. The wells of a sterile 96-well flat-bottomed polystyrene plate were filled with 100 µL of BHI medium containing 1:100 dilution of overnight bacterial culture. The plates were incubated for 24 h at 37°C in static condition, then the content of the plates was poured off and the wells were washed to remove the unattached bacteria. 100 μL of fresh BHI containing or not containing KHS-Cnd at MIC concentration were added into each well. The plates were incubated for additional 24 h (48 h in total) at 37°C. At the end the plates were analyzed as described above. Each data point is composed of three independent experiments, each performed in at least three replicates.

### 2.6 Motility assays

#### 2.6.1 Twitching and surface-associated motility

A single colony of *A. baumannii* was inoculated in 5 mL of Nutrient Broth (Oxoid, Basingstoke, UK) in a sterile conical bottom tube and incubated overnight at 37°C under constant stirring at 180 rpm. The semisolid medium used for twitching and surface-associated motilities was prepared with 0.5% Tryptone (Oxoid, Basingstoke, UK), 0.25% sodium chloride (Sigma, Steinheim, Germany), and 0.3% agarose (Invitrogen, Paisley, UK). After autoclaving, the medium was deposited into 6-well polystyrene plates and allowed to solidify. The analyses were conducted in the presence and in the absence of ¼ KHS-Cnd MIC value. The peptide was dissolved in the medium before agar solidification. For the study of twitching motility, 2 µL of overnight bacterial culture were inoculated on the bottom of the well (between the semisolid medium and the plastic) while, for investigating surface-associated motility, 2 µL of overnight inoculum were inoculated on the surface of the semisolid medium. Subsequently, the plates were incubated at 37°C and motility was analyzed at 24 and 48 h (Corral et al., 2021).

### 2.7 Synergism assay

The synergism between the KHS-Cnd peptide and ceftazidime/avibactam (CZA) was evaluated by the checkerboard assay in *A. baumannii* strains resistant to this antibiotic. Double serial dilutions of KHS-Cnd and CZA were tested in combination in a sterile 96-well polystyrene flat-based plate. The peptide was tested at concentrations ranging from 20 µM to 0.156 µM and CZA was tested at concentrations ranging from 128-32 µg/mL to 8-2 µg/mL, respectively. All determinations were performed in triplicate.

To classify the antimicrobial action of two molecules in combination, the fractional inhibitory concentration (FIC) or synergy index was calculated as reported below (Zhang et al., 2024):


$$FIC=FICa+FICb=\frac{MICA}{MICa}+\frac{MICB}{MICb}$$

In the formula MIC A and MIC B are the minimum concentrations of two tested compounds inhibiting bacterial growth when used in combination, and MICa and MICb are the MICs of the two antimicrobials used individually. If the obtained FIC value is <0.5 the effect of the two molecules is considered synergistic, if the value is between 0.5 and 4 the combination is indifferent, if it is > 4.0 they have an antagonistic effect.

### 2.8 Eukaryotic cells

The adenocarcinomic human alveolar basal epithelial cells A549 (ATCC CRM-CCL-185) were cultured in medium with: DMEM high glucose (4.5 g/L), sodium pyruvate without L-glutamine, 10% fetal bovine serum (FBS), 1% glutamine, and 1% penicillin–streptomycin in an atmosphere of 5% CO_2_ at 37°C. All media were from Euroclone (Milan, Italy). Confluent monolayers were used 24 h after seeding.

### 2.9 Antibiotic protection assay

1,25 × 10^5^ cells/well of A549 cells were cultured at 37°C and 5% CO_2_ in 24-well plates (BD Falcon, NY, USA) with a medium containing: DMEM, 10% FBS, 1% glutamine with 1% antibiotic (penicillin-streptomycin) until complete confluence (usually after 24 h of incubation). Two hours before infection, the culture medium was replaced with basal medium containing: DMEM medium plus 1% glutamine without FBS and antibiotics. Clinical strain Ab12 was grown overnight in BHI broth at 37°C at 180 rpm. Bacteria were diluted 1:100 in BHI and sub-cultured to 0.5 OD/mL (600 nm) at 37°C in the presence and absence of 2.5 μM KHS-Cnd. Then, human cells were infected separately with the KHS-Cnd-treated or untreated bacterial suspension at a multiplicity of infection (MOI) of about 100 bacteria for eukaryotic cell (MOI 1:100) for 3 h at 37°C in 5% CO_2_ (Jiang et al., 2024).

After 3 h, unbound bacteria were removed from the cell monolayers by two washes with 2 mL of PBS. Then, the cells were lysed with 0.025% Triton X-100, serially diluted, and plated on Tryptone soy agar (Oxoid, UK) to count the viable adherent and internalized bacteria.

To determine the internalized bacteria, the cell monolayers were washed with 2 mL of PBS; then, 500 μL of fresh medium containing 200 µg/mL of gentamicin was added to each well and incubated in the same conditions for 1 h to kill the bacteria adhering to cells. The sensitivity of bacteria to gentamicin and the absence of toxicity toward A549 cells was previously verified. After this additional hour, cells were lysed with 0.025% Triton X-100 and the collected supernatants were plated on Tryptone soy agar (Oxoid, UK), followed by overnight incubation at 37°C to count the viable intracellular bacteria. Data represent the mean of four independent experiments. Adhesion is expressed as CFU of bacteria that adhered to A549 cells 1 h post-infection at 37°C. Invasion efficiency is expressed as the CFU of bacteria that were gentamicin resistant 1 h post-infection.

### 2.10 Statistical analysis of biological evaluation

Data were statistically validated using Student’s T-test comparing experimental data of treated and untreated samples. The significance of differences between mean absorbance values was calculated using a two-tailed Student’s T-test.

## 3 Results

### 3.1 Antimicrobial resistance profile and phenotypic characterization of clinical and reference *Acinetobacter baumannii strains*

*A. baumannii strains* were classified according to their antimicrobial resistance profile. As reported in **Table 1**, we tested ten antibiotics, eight belonging to four different classes of drugs, as reported in the guidelines of the “European Committee on Antimicrobial Susceptibility Testing 2023” (EUCAST) for the genus *Acinetobacter* spp, the remaining two classified in a miscellaneous class. Susceptible strains have been classified as Wild-type (WT); strains resistant to at least one antimicrobial drug belonging to three or more antibiotic classes were reported as multidrug-resistant (MDR); strains resistant to all antimicrobials of all classes were defined as pandrug-resistant (PDR).

**Table 1:** Antimicrobial resistance profiles of reference and clinical *A. baumannii* strains.

Strains	Cephalosporin	Carbapenem		Fluoroquinolone		Aminoglycoside			Miscellaneous		
	CZA	MRP	IM	CIP	LEV	AK	CN	TOB	SXT	COL	
	8-2 mg/L	10μg	10μg	5μg	5μg	30μg	10μg	10μg	(25μg)	S ≤ 2mg/L	
**17978**	S	S	S	I	S	S	S	S	I	S	**WT**
**19606**	S	S	S	I	S	R	R	R	R	S	**MDR**
**Ab1**	R	R	R	R	R	S	R	R	R	S	**PDR**
**Ab2**	R	R	R	R	R	S	R	R	R	S	**PDR**
**Ab3**	R	R	R	R	R	R	R	R	R	S	**PDR**
**Ab4**	R	R	R	R	R	R	R	R	R	S	**PDR**
**Ab11**	R	S	S	S	S	S	S	S	S	S	**WT**
**Ab12**	S	S	S	S	S	S	S	S	S	S	**WT**

Antibiotic resistance profile was tested according to the guidelines of EUCAST Clinical Breakpoint Tables v. 13.0 (valid from 1 January 2023). CZA, ceftazidime/avibactam; MRP, meropenem; IM, imipenem; CIP, ciprofloxacin; LEV, levofloxacin; AK, amikacin; CN, gentamicin; TOB, tobramycin; SXT, trimethoprim/sulfamethoxazole; COL, colistin; S, sensitive; R, resistance; I, intermediate; WT, sensitive strain; MDR, multidrug resistant; PDR, pandrug resistant.

Phenotypic features of clinical and reference strains are summarized in **Table 2**. All bacterial strains were able to form biofilm with different capabilities, independently on their antimicrobial profile. According to Biofilm Formation Index (BFI) strains were classified as weak (0.1> BFI≤ 0.5), moderate (0.5> BFI≤ 1) and strong (BFI>1) biofilm producers (Cangui-Panchi et al., 2022). As expected, the biofilm production resulted higher after 48h of bacterial growth. In particular, strongest biofilm former strains are clinical isolates Ab1, Ab2 (both PDR strains), Ab11 and Ab12 (both WT strains). Only two strains (ATCC17978 and Ab12) showed surface-associated motility. Ab1, Ab2 and Ab12 strains also possessed twitching motility.

**Table 2:** Phenotypic features of *A. baumannii* reference and clinical strains.

Bacterial Strain	Biofilm 24 ha (OD 590 nm)	Biofilm Formation Index 24 h (BFI)	Biofilm 48 h^b^ (OD 590 nm)	Biofilm Formation Index 48 h (BFI)	Surface-associated Motility	Twitching
ATCC 17978	0.473 ± 0.044	weak	0.312 ± 0.061	weak	+	–
ATCC 19606	0.595 ± 0.027	moderate	0.746 ± 0.057	moderate	–	–
Ab1	0.662 ± 0.129	moderate	2.397 ± 0.451	strong	–	+
Ab2	1.656 ± 0.160	strong	2.785 ± 0.094	strong	–	+
Ab3	0.242 ± 0.064	weak	0.611 ± 0.159	moderate	–	–
Ab4	0.498 ± 0.089	weak	0.732 ± 0.196	moderate	–	–
Ab11	0.687 ± 0.099	moderate	1.344 ± 0.251	strong	–	–
Ab12	1.057 ± 0.081	strong	1.423 ± 0.145	strong	+	+

^a^Biofilm formation after growth for 24 h without medium replacement. ^b^Biofilm formation after growth for 48 h with medium replacement after 24 h.

### 3.2 Effect of KHS-Cnd on bacterial viability

Preliminary experiments were performed to evaluate the antimicrobial activity of KHS-Cnd peptide on planktonic growth of *A. baumannii* (**Table 3**). The obtained results showed that the peptide inhibits viability of bacterial strains at a concentration ranging between 5 µM and 10 µM (MIC). The bactericidal activity (MBC) was always observed at a double concentration compared to the MIC value, except for strains Ab11 and Ab12 where minimal inhibiting and minimal bactericidal concentration coincide.

**Table 3:** Minimal inhibitory (MIC) and bactericidal (MBC) concentration of KHS-Cnd peptide on *A. baumannii* strains.

Strains	MIC (µM)	MBC (µM)
ATCC 17978	10	20
ATCC 19606	5	10
Ab 1	5	10
Ab 2	10	20
Ab 3	5	10
Ab 4	5	10
Ab 11	10	10
Ab 12	10	10

Considering these results, experiments conducted to search for a possible anti-virulence activity were performed testing 1/4 MIC values of KHS-Cnd.

### 3.3 Antibiofilm activity of KHS-Cnd peptide on *A. baumannii* strains

**Figure 1:**
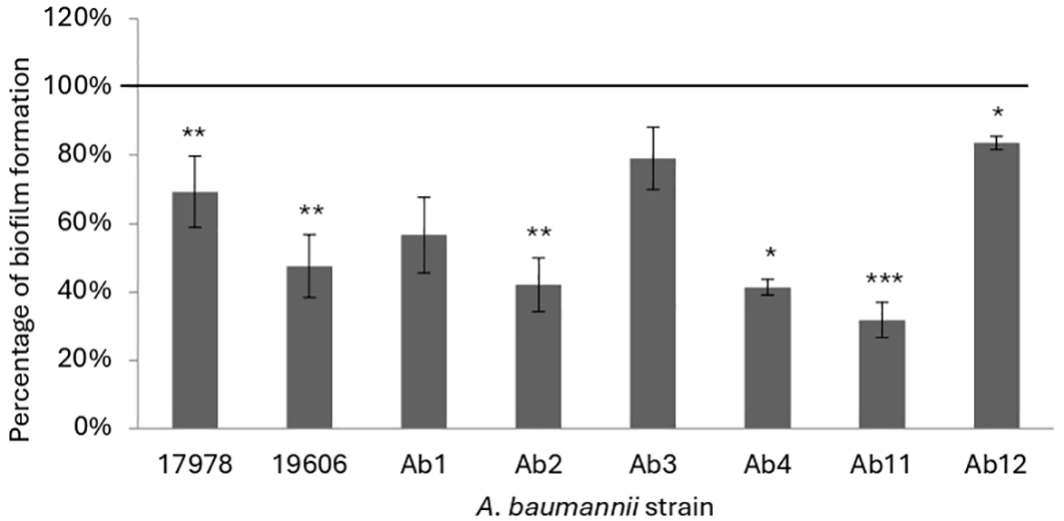
Effect of KHS-Cnd on biofilm formation of *A. baumannii* strains. In each bacterial strain KHS-Cnd was used at a concentration equivalent to ¼ of the corresponding MIC value. In the ordinate axis, the data are expressed as the percentage of biofilm formed in presence of KHS-Cnd compared with that of untreated bacteria. Each data point is composed of three independent experiments, each performed at least in three replicates. Statistical difference was determined by Student’s *t*-test: *p < 0.05; **p < 0.01; ***p < 0.001 compared with the control.

The antibiofilm activity of KHS-Cnd was studied both before adhesion of bacterial cells, by adding the peptide to the medium at time zero, and on the mature biofilm, when the peptide was added after 24 h of bacterial growth. For each experiment bacteria were also grown in BHI medium without the peptide as a control. The results of the KHS-Cnd addition on the pre-adhesion period are shown in **Figure 1**. Results were expressed as the percentage of biofilm formed in presence of KHS-Cnd compared to untreated bacteria using a concentration corresponding to the ¼ of MIC value. As reported in **Figure 1**, results showed that biofilm formation was inhibited by KHS-Cnd for all tested strains, with an inhibition percentage ranging between 10% and 65%. The reduction in biofilm formation was statistically significant in six out of eight tested strains. Strongest inhibition was obtained on the clinical isolates Ab11, Ab 2 and Ab4 and on the reference strain ATCC 19606.

**Figure 2:**
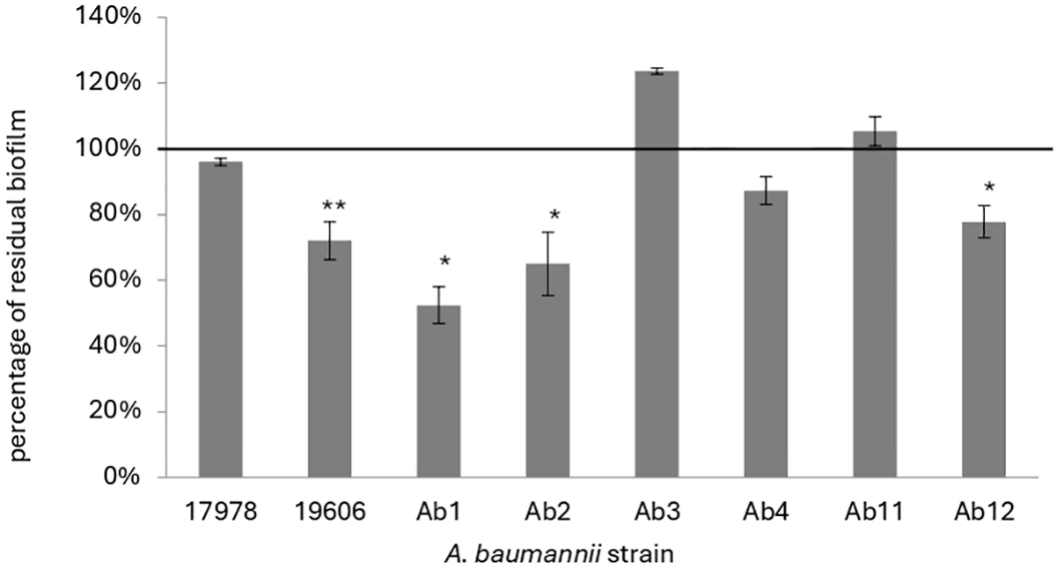
Effect of KHS-Cnd used at MIC values on mature biofilm of *A. baumannii*. The percentage of residual biofilm is shown on the ordinate axis. Data are expressed as percentage of residual biofilm after 24 h of treatment with KHS-Cnd compared to untreated control sample. Each data point is composed of three independent experiments, each performed at least in three replicates. Error bars indicate the standard deviations of all measurements. Statistical difference was determined by Student’s t-test: *p < 0.05; **p < 0.01 compared with the control.

Afterwards the effect of KHS-Cnd was evaluated on mature biofilm by adding the peptide after 24 h of bacterial sessile growth. Since mature biofilm disaggregation is very hard to obtain, we chose a peptide concentration corresponding to the MIC value. A statistically significant reduction of preformed biofilm, ranging between approximately 20% and 50% was observed in four out of eight tested strains (**Figure 2**). Although these data are less impactful than those obtained on early phases of biofilm formation, it is worth noting that biofilm measured after 48 h of incubation was more abundant, undoubtedly more structured, and difficult to eradicate. These results suggest that the action of KHS-Cnd peptide is not limited to the initial bacterial adhesion on an abiotic surface but is also effective on preformed biofilm.

### 3.4 Effect of KHS-Cnd on *A. baumannii* motility

We analyzed also the effect of KHS-Cnd on twitching and surface-associated motility of *A. baumannii* strains after 24 h and 48 h of treatment.

**Figure 3:**
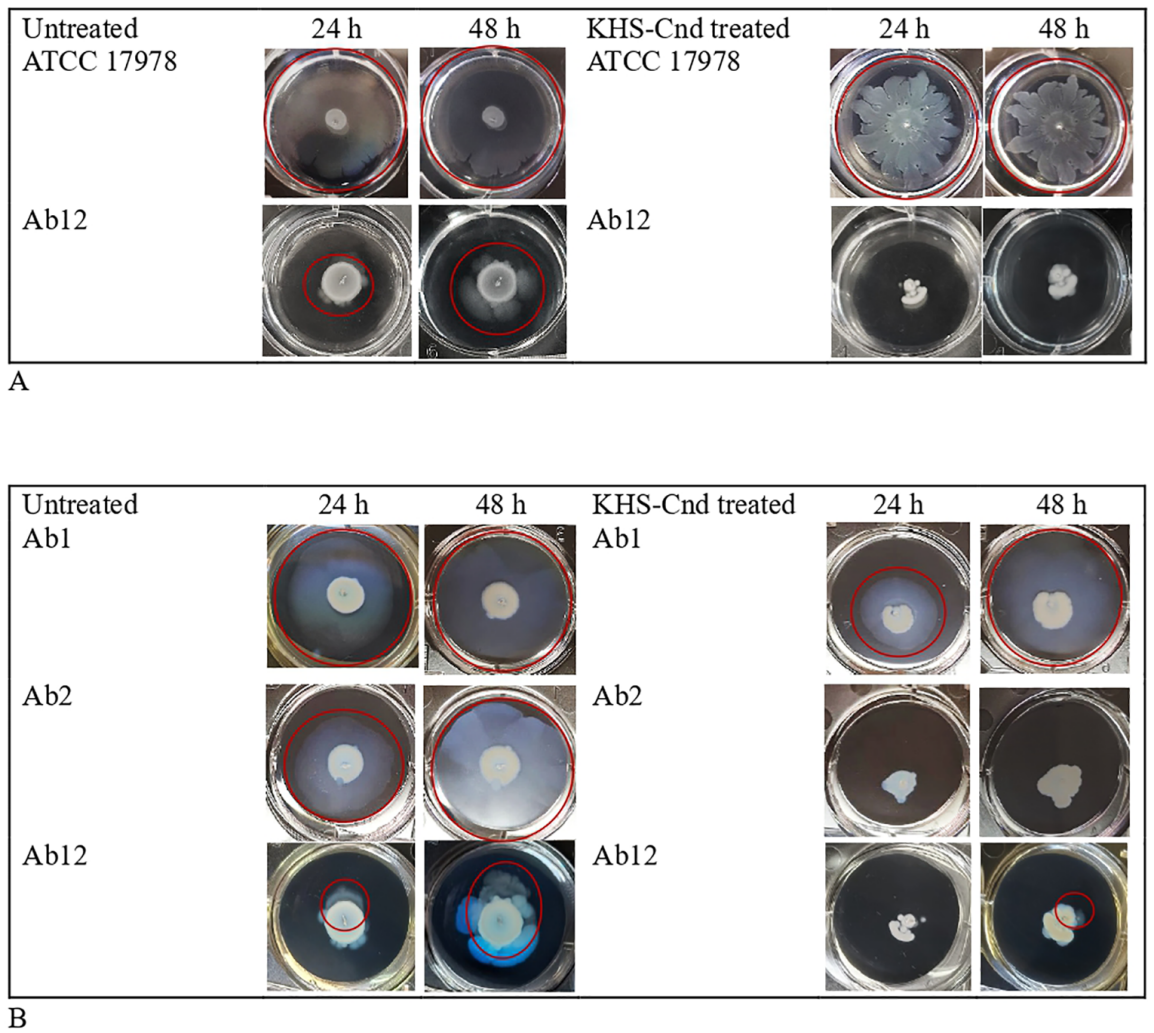
Effect of KHS-Cnd on *A. baumannii* motility. **(A)** Surface-associated motility of *A. baumannii* bacterial strains untreated (left panel) and KHS-Cnd treated (right panel) photographed at 24 h and 48 h Red circles highlighted the bacterial motility. **(B)** Twitching assay of *A. baumannii* bacterial strains untreated (left panel) and KHS-Cnd treated (right panel) photographed at 24 h and 48 h Red circles highlighted the bacterial motility.

The surface-associated motility was analyzed on the surface of a semi-solid medium (medium/air interface), while twitching motility was analyzed on bacteria moving between the bottom of the polystyrene plate and the semi-solid medium. The study of the two different motilities was conducted simultaneously using the same well and medium (**Figure 3**). Furthermore, both motilities were observed after incubating the plates for 24 h and 48 h. Peptide concentrations used in this experiment were the same of the treatment of early biofilm formation (1/4 MIC value). The images of the surface motility are shown in **Figure 3**.

Only strains ATCC 17978 and Ab12 have shown a surface-associated motility, as observed in the left panel of **Figure 3A** (see the red circle).

The reference strain ATCC 17978 already showed greatest surface-associated motility after just 24 h of incubation, while for the clinical strain Ab12, it was better observed after 48 h of incubation. KHS-Cnd showed an inhibitory effect on the motility of both strains, with a more marked effect on the clinical strain Ab12 (**Figure 3A**).

Twitching motility, highlighted by a red circle in the images shown in **Figure 3B**, was measured both with and without KHS-Cnd used at a concentration of 1/4 MIC. Indeed, by placing the plates against the light, it was possible to observe a specific halo produced by bacteria moving in eccentric directions between the bottom of the wells and the semi-solid medium.

After 24 h of peptide treatment, a light decrease in twitching motility was observed in Ab1 strain. The effect was completely lost after 48 h of incubation. Conversely, in strain Ab2 a total absence of twitching motility was observed after KHS-Cnd treatment, both after 24 and 48 h of incubation. Lastly, a complete absence of twitching motility was found following KHS-Cnd treatment on Ab12 strain after 24 h of incubation. After 48 h of bacterial growth, instead, a small halo was observed on the bottom of the semi-solid medium, which suggests a partial effect of KHS-Cnd on this strain at this time point.

### 3.5 Synergistic effect of KHS-Cnd and ceftazidime/avibactam

To test a possible synergism between the antimicrobial peptide KHS-Cnd and ceftazidime/avibactam (CZA), these two compounds were added to the resistant strains Ab1, Ab2, Ab3, Ab4 and Ab11.

Based on the FIC values reported in **Table 4**, the combination of KHS-Cnd with ceftazidime/avibactam was found to be indifferent in terms of antibacterial activity for all the selected clinical strains.

**Table 4:** Synergistic effect of KHS-Cnd and ceftazidime/avibactam on *A. baumannii* clinical resistant strains.

Strain	MIC CZA	MIC KHS-Cnd	CZA + KHS-Cnd		FIC
	(mg/L)	(µM)	CZA	KHS-Cnd	
			(mg/L)	(µM)	
Ab1	64-16	5	32-8	2.5	1
Ab2	64-16	10	32-8	5	1
Ab3	64-16	5	32-8	2.5	1
Ab4	64-16	5	16-4	2.5	0.75
Ab11	64-16	10	32-8	5	1

In particular, the FIC obtained values demonstrate the absence of a synergistic or antagonistic effect between the antibiotic and the antimicrobial peptide. However, in the presence of KHS-Cnd, MIC values of ceftazidime/avibactam are on average halved and, in the case of Ab4 they were 4-fold lower (**Table 4**).

### 3.6 Effect of KHS-Cnd on adhesion and invasion of *A. baumannii* to eukaryotic cells

The anti-virulence activity of the KHS-Cnd peptide was also evaluated on the ability of *A. baumannii* to adhere to and invade human lung epithelial cells. For this assay, A549 human alveolar basal epithelial cells were used, since Ventilator-Associated Pneumonia (VAP) is one of the most frequent infections in which *A. baumannii* is involved. Bacterial resistance to gentamicin was evaluated, and the clinical strain Ab12 was the only one sensitive to this antibiotic, at a concentration of 200 µg/mL. Since, as reported in literature, *A. baumannii* does not have high invasive abilities, we decided to use a multiplicity of infection (MOI) of 1:100. Adhesion and invasion efficiency of bacteria treated and untreated with KHS-Cnd peptide are shown in **Table 5**. Adhesion was defined by the number of bacteria adhering to A549 cells after 3 h of incubation in the presence and absence of KHS-Cnd. To obtain the bacteria internalized in A549 cells, these were treated for 1 h with gentamicin (200 µg/mL) to kill the external bacteria attached to them. The adhesion efficiency of both treated or untreated Ab12 strain corresponded to approximately 1.5% of the total CFU used (about 3 x 10^8^ bacterial cells). Therefore, our results showed that the adhesion efficiency of *A. baumannii* strain Ab12 was not affected by KHS-Cnd incubation.

**Table 5:** The adhesion and invasion capabilities of *A. baumannii* Ab12 on A549 cells in the presence and absence of 1.25 µM KHS-Cnd.

	Untreated		KHS-Cnd treated	
	Adhesion	Invasion	Adhesion	Invasion
**CFU**	4.76x10^6^ ± 0.03x10^6^	7.42x10^3^ ± 0.97x10^3^	5.30x10^6^ ± 0.06x10^6^	5.00 x10^3^ ± 0.27x10^3^
**Percentage**	1.50% ± 0.01% **^a^**	0.15% ± 0.02% **^b^**	1.50% ± 0.02% **^a^**	0.09 ± 0.00% **^b^**

Data represent the mean ± SD of four independent experiments. CFU, colony-forming unit.

^a^Percentage of adhered bacteria to A549 cells compared to the total CFU used in the experiment.

^b^Percentage of bacteria able to invade the cells compared to the total adhered bacteria.

Approximately 0.15% of the total bacteria adhering to A549 cells were able to invade host cells (approximately 10^3^ bacteria) (**Table 5**). Interestingly, the invasion efficiency was reduced after treatment with 1.25 µM KHS-Cnd, corresponding to 1/4 MIC. Furthermore, the results were statistically significant with a *p* value of 0.024153.

## 4 Discussion

Due to its high resistance to antibiotics, WHO has designated *A. baumannii* in the list of ESKAPE pathogens as a priority pathogen, for which it is urgent and necessary to identify innovative therapeutic therapies. The ability to develop a biofilm poses a severe challenge to the clinical management of *A. baumannii* infections. These latter are frequently associated with the use of catheters in hospital settings (Rajkumari and Siddhardha, 2020) and have been related to significant mortality and morbidity. Moreover, the formation of biofilm is strictly related to the development of antibiotic resistance (Gedefie et al., 2023).

Our study is focused on the assessment of antibacterial and anti-virulence activity exerted on *A. baumannii* by the peptide KHS-Cnd, a mutant derived from the natural chionodracine (Cnd) peptide identified in the Antarctic fish *Chionodraco hamatus*.

To this aim, the antimicrobial profiles were analyzed in selected *A. baumannii* strains, six clinical strains (four PDR and two WT strains) and two reference strains (one MDR and the other WT).

All clinical isolates form biofilm with different capability, regardless of whether the strain is drug resistant or sensitive. Biofilm represents a phenotypic mode of resistance to the action of antibiotics due to a multiplicity of mechanisms, including also the presence of the extracellular matrix acting as a mechanical barrier to their penetration. Furthermore, biofilm favors the exchange of genetic material between bacterial cells, thus supporting the onset of WT phenotype mutations in MDR and PDR strains.

Conventionally, antimicrobials have been employed also to obtain biofilm control and impair bacterial growth. However, this approach is not decisive and conversely can bring an increased bacterial resistance (Varela et al., 2021). AMR is an alarming and ever-growing phenomenon. One of the most exploited strategies to eradicate MDR or PDR pathogens is combination therapy, based on the combined use of two or more antibiotics to treat an infection against which one or both drugs are ineffective.

Moreover, recent research has highlighted antimicrobial peptides (AMPs) as biofilm inhibitors capable of interfering with development mechanisms, leaving bacterial growth unaffected (de Moura Cavalheiro et al., 2024). The antibiofilm effects of AMPs act on several factors, including membrane permeability, compactness of extracellular polymeric matrix, cell adhesion and attachment to the substrate (Artini et al., 2022; Guo et al., 2024). Furthermore, the synergism between an antibiotic against which the pathogen has developed resistance and an anti-virulence molecule could allow the recovery of the antibiotic effectiveness.

Our main goal was to identify an AMP that could influence the virulence features of *A. baumannii* rather than its bacterial viability. Considering a previous work where we assessed the ability of different AMPs to kill ESKAPE pathogens (Olivieri et al., 2018) and a following paper investigating the capacity of one of these peptides, named KHS-Cnd, to affect biofilm formation of a MDR *Pseudomonas aeruginosa* clinical strain (Artini et al., 2022), we decided to test this bioactive molecule also on the six *A. baumanni* clinical isolates and two reference strains. KHS-Cnd represents a promising candidate as it displayed a low cytotoxicity against human primary cells, a low hemolytic activity, but a significantly high bactericidal activity against drug-resistant *Enterococcus faecium*, *Staphylococcus aureus*, *Klebsiella pneumoniae*, *A. baumannii*, *Pseudomonas aeruginosa* and *Enterobacter* sp. KHS-Cnd exhibited high antibacterial activity against all tested bacterial species, particularly against Gram-negative species (Olivieri et al., 2018; Imperlini et al., 2024).

Moreover, KHS-Cnd also exerts an anti-virulence potential as we demonstrated on five *P. aeruginosa* clinical isolates from cystic fibrosis patients. KHS-Cnd, in fact, affected biofilm development and caused biofilm disaggregation. The peptide was also able to reduce adhesion to pulmonary cell lines and invasion of host cells by *P. aeruginosa* (Artini et al., 2022).

In this paper, we first determined that the KHS-Cnd peptide is antimicrobial at concentration ranging from 5 to 10 µM against the six clinical and two reference *A. baumannii* strains. Moreover, we evidenced that it showed a significative antibiofilm activity on all tested strains at sub-inhibitory concentrations. In addition, its activity is not only restricted to biofilm formation but also on mature biofilm on five out of eight tested *A. baumannii* strains. Therefore, these results strengthen and confirm the excellent abilities of this promising drug candidate to interfere with bacterial adhesion.

Other AMPs, both natural and synthetic, have been reported to display antimicrobial and antibiofilm activity against *A. baumannii* (Sharma et al., 2019; Park et al., 2022; Farzi et al., 2024; Alexander et al., 2024; Girdhar et al., 2024). The naturally occurring host defense LL-37 peptide, a human cathelicidin known for its antimicrobial properties, showed antibacterial efficacy against *A. baumannii* with a MIC value of 32 µg/mL and anti-biofilm activity at 1/4 × MIC and 1/2 × MIC concentrations (Farzi et al., 2024). A 12-residue synthetic self-assembled peptide, instead, named SA4, showed antibacterial action against *A. baumannii* with MIC value ranging between 50 µg/mL and 100 µg/mL and prevented the growth of mature bacterial biofilms (Sharma et al., 2019). Moreover, hylin peptides isolated from the electro-stimulated arboreal South American frog *Hypsiboas albopunctatus* exhibited broad-spectrum antimicrobial activity and anti-biofilm activity against carbapenem-resistant *A. baumannii* (Park et al., 2022). Lynronne AMPs from rumen microbioma showed antimicrobial and anti-biofilm activity towards *A. baumannii* (MIC 2-128 μg/mL). Lynronne-2 and -3 demonstrated additive effects with amoxicillin and erythromycin, and synergy with gentamicin (Alexander et al., 2024). It is worth noting that the MIC values of KHS-Cnd we determined (5-10 µM corresponding to 12.5-25 µg/mL) are lower than those reported for the mentioned AMPs. This difference is still more marked if we consider that 1/4 of MIC value was the KHS-Cnd concentration needed of KHS-Cnd to inhibit biofilm formation and disaggregate mature biofilm.

Biofilm formation is a multifactorial dynamic process, typically characterized by different phases: reversible adhesion of bacteria, formation of microcolonies characterized by matrix secretion that renders bacterial adhesion irreversible, biofilm maturation with the development of a three-dimensional structure and detachment of bacterial cells from biofilm that revert to planktonic phenotype and disperse away to colonize new sites (Mea et al., 2021). The initial adhesion to the surfaces happens via appendages like pili, fimbriae or flagella, sometimes associated to a swarming motility. Although *A. baumannii* has long been defined as non-motile, it has been demonstrated that it possesses two kinds of motilities defined as surface-associated and twitching motility (Jeong et al., 2024).

Twitching motility is a coordinated multicellular movement caused by the extension, attachment, and retraction of type IV pili, which allows *A. baumannii* motion between a liquid medium and an abiotic surface such as polystyrene. It is involved in surface adherence and in the early stages of biofilm formation. Conversely, surface-associated motility is not associated to appendages, but seems most likely driven by the release of extra polymeric molecules.

Several studies have indicated that motility plays a key role in the development of *A. baumannii* infections (Jeong et al., 2024; Corral et al., 2021). For this reason, we also investigated the effect of KHS-Cnd on motility.

Our results showed that only two strains (ATCC 17978 and clinical Ab12) possessed surface-associated motility; twitching motility, instead, was present in clinical Ab1, Ab2 and Ab12 strains. Surface-associated motility was strongly impaired by KHS-Cnd treatment for both strains. Twitching motility was completely inhibited by KHS-Cnd in Ab2 strain, while in Ab1 and Ab12 strains the inhibition was particularly evident only at 24 h of incubation.

Similarly, the proline-rich antibacterial peptide Bac7 has been reported to reduce the twitching motility and biofilm formation of *A. baumannii* without inducing resistance at sub-inhibitory concentrations (Dolzani et al., 2019). Also the AMP named Cec4, belonging to the cecropin family in *Musca domestica*, exhibited both antibacterial and anti-biofilm properties against a carbapenem resistant strain of *A. baumannii* (Liu et al., 2020); in particular, Cec4 peptide interfered with the production of type IV pilus assembly proteins and reduced *A. baumannii* motility (Liu et al., 2020).

KHS-Cnd was also unable to reduce the adhesion of *A. baumannii* to a biotic substrate such as pulmonary cell lines. Contrary to expectations, the invasion of host cells by *A. baumannii* was significantly impaired after treatment with a reduction around 32% at a concentration of 3.125 µg/mL (1.25 µM) of KHS-Cnd. It is worth noting that the invasion is not a predominant virulence factor in *A. baumannii* infection.

Other molecules, such as ceragenins, cholic acid-based compounds that imitate the action of AMPs, showed an inhibitory effect on the adhesion of *A. baumannii* to A549 cell line. This activity was dose-dependent with most prominent effect at 10 μg/mL (Karasiński et al., 2024).

Our data therefore highlighted that the antibiofilm properties of KHS-Cnd on *A. baumannii*, biofilm disruption without bacterial killing, could facilitate the further dissemination of the pathogen. A feasible solution to obtain both results could be the administration of KHS-Cnd in synergy with conventional antibiotics. Therefore, we investigated the potential synergistic effects of the peptide with ceftazidime/avibactam. Despite FIC values did not show a clear synergistic activity between the antibiotic and the antimicrobial peptide, in the presence of KHS-Cnd, MIC values of ceftazidime/avibactam were notably lower.

Interestingly, it has been reported that frog-skin AMP, namely Esc, used in combination with colistin against multidrug-resistant *A. baumannii* clinical isolates, has a synergistic activity in inhibiting the growth and killing of tested strains (Sacco et al., 2022). This effect was probably due to the membrane-perturbation operated by colistin and AMP, both acting on bacterial membrane (Sacco et al., 2022). Ceftazidime, on the contrary, exerts its bactericidal mechanism by binding of penicillin binding protein (PBP) and inhibition of cell wall synthesis, suggesting that the synergistic effect with KHS-Cnd is not merely due to a disturbance in membrane integrity.

In conclusion, this research adds novel evidence about the antibiofilm properties of KHS-Cnd peptide and its ability to attenuate the virulence of ESKAPE pathogens although without impacting bacterial growth. To date, anti-virulence studies have been conducted only on two species belonging to the ESKAPE group. Therefore, it would be interesting to expand the analysis to include the others as well. These findings could be useful to identify new drugs against *A. baumannii* infections and underline the importance of the AMPs as “adjuvants” of the conventional therapy when they exhibit a synergistic effect if administered together with conventional antibiotics. The possibility to employ, in this case, sub-MIC concentrations of the peptide is fundamental to reduce its potential toxicity and, at the same time, the costs of this innovative therapy. For this reason, in the current study, the synergistic action between the peptide and ceftazidime/avibactam was tested. Due to the interesting results, it would be worthwhile to expand the study to include other antibiotic classes and a wider range of multidrug-resistant strains. Additionally, further research into formulations, like liposomes or nanoparticles, that enhance the effectiveness and stability of the peptide will be essential.

Furthermore, comprehensive studies are needed to better understand the molecular mechanisms behind the antibiofilm and virulence attenuation properties observed, as well as to explore the physiological basis of this phenomenon in major detail.

## Data Availability

The original contributions presented in the study are included in the article/supplementary material. Further inquiries can be directed to the corresponding author.
